# Supplementary material for: Deletion of wfs1 Impairs Oligodendrocyte Precursor Cells Dorsal Distribution and Myelination Through the wfs1-hmgcs1 Axis in Zebrafish
Source: Biology (Basel). 2026 Aug 16;15(16):1402. doi: 10.3390/biology15161402 (PMC13510151; doi:10.3390/biology15161402)
Supplement: Supplementary file 1 [file biology-15-01402-s001.zip › File S1-Transcriptome sequencing data and differentially expressed genes (DEGs) in wfs1-- zebrafish at 5 dpf/2.KEGG/wfs1KOvsWT/UP/plot/wfs1KOvsWT.html]

### wfs1KOvsWT KEGG通路富集列表

---

```
## Warning: package 'knitr' was built under R version 3.5.1
```

| KEGGID | Description | GeneRatio | BgRatio | pvalue | padj | geneID | geneName | keggID | Count |
| --- | --- | --- | --- | --- | --- | --- | --- | --- | --- |
| dre04672 | Intestinal immune network for IgA production | 8/511 | 37/8379 | 0.0014498 | 0.2218189 | 799354/30762/368878/30763/571282/100006078/100151365\_1/100333914 | cxcl12b/mhc2dab/si:busm1-266f07.2/mhc2dcb/LOC571282/ccr9b/si:dkey-1h24.6/ccl25a | dre:799354/dre:30762/dre:368878/dre:30763/dre:571282/dre:100006078/dre:100151365/dre:100333914 | 8 |
| dre00270 | Cysteine and methionine metabolism | 11/511 | 73/8379 | 0.0044206 | 0.3216465 | 335715/334195\_1/103911233/317744\_1/561410\_1/445818/431773\_1/30659\_1/103911241/563642/450084\_1 | mdh1ab/mat1a/LOC103911233/dnmt3bb.1/tat/cthl/enoph1/dnmt3bb.2/LOC103911241/sdsl/gss | dre:335715/dre:334195/dre:103911233/dre:317744/dre:561410/dre:445818/dre:431773/dre:30659/dre:571201/dre:563642/dre:450084 | 11 |
| dre04130 | SNARE interactions in vesicular transport | 9/511 | 56/8379 | 0.0063068 | 0.3216465 | 567385\_1/449923/436605/449923\_1/393836/571872/325484/393236/565822\_1 | vti1a/si:ch73-234b20.5/stx5a/si:ch73-234b20.5/stx11b.1/zgc:165520/vamp5/sybl1/bnip1a | dre:567385/dre:449923/dre:436605/dre:449923/dre:393836/dre:571872/dre:325484/dre:393236/dre:565822 | 9 |
| dre01200 | Carbon metabolism | 19/511 | 173/8379 | 0.0089859 | 0.3437100 | 335715/560753\_1/116517/445233/560827\_1/406762\_1/403070\_1/437019\_1/541329/570579\_1/100006589\_1/550579\_1/561416\_1/317746/406702/563642/321224/751668/568001\_3 | mdh1ab/tpi1b/adh5/me2/prps1b/pgd/rgn/pcca/gcsha/g6pd/idh1/idh3g/pfkpb/suclg2/pdha1a/sdsl/hkdc1/gck/pfkmb | dre:335715/dre:560753/dre:116517/dre:445233/dre:560827/dre:406762/dre:403070/dre:437019/dre:541329/dre:570579/dre:100006589/dre:550579/dre:561416/dre:317746/dre:406702/dre:563642/dre:321224/dre:751668/dre:568001 | 19 |
| dre03018 | RNA degradation | 13/511 | 108/8379 | 0.0139295 | 0.4262429 | 373085\_1/448949\_1/394064\_1/553665\_1/100538016/492479\_1/565000/561416\_1/317641\_1/566017\_1/565659\_1/321035\_1/568001\_3 | hspa9/cnot1/exosc10/cnot6l/si:dkey-79d12.5/btg3/exosc3/pfkpb/zcchc7/lsm8/edc4/pabpc4/pfkmb | dre:373085/dre:448949/dre:394064/dre:553665/dre:100538016/dre:492479/dre:324574/dre:561416/dre:317641/dre:566017/dre:565659/dre:321035/dre:568001 | 13 |
| dre03440 | Homologous recombination | 7/511 | 46/8379 | 0.0203144 | 0.4496866 | 558923/406487/404608/794038/406721/554171\_1/450081 | palb2/rad51/rad51d/brip1/rad51b/rad52/rad51c | dre:558923/dre:406487/dre:404608/dre:794038/dre:406721/dre:554171/dre:450081 | 7 |
| dre00590 | Arachidonic acid metabolism | 9/511 | 72/8379 | 0.0300955 | 0.4496866 | 406449\_1/494108/559020/393297/406370/566996/406575\_1/393592/560649 | ptges3a/ptgr1/ptgs2b/zgc:56585/pla2g6/si:dkey-11k2.7/lta4h/cyp2p9/si:ch211-214p16.3 | dre:406449/dre:494108/dre:559020/dre:393297/dre:406370/dre:566996/dre:406575/dre:393592/dre:560649 | 9 |
| dre00470 | D-Amino acid metabolism | 3/511 | 12/8379 | 0.0328172 | 0.4496866 | 560387/405800/619259 | c17h14orf159/dao.2/dao.1 | dre:560387/dre:405800/dre:619259 | 3 |
| dre02010 | ABC transporters | 8/511 | 63/8379 | 0.0365538 | 0.4496866 | 368620\_1/570453\_1/406803\_1/406803\_2/571189/570148\_1/735310/556826\_1 | abcc4/abcg2b/abcd3a/abcd3a/abcb11b/abcb9/abcg2d/si:dkey-57h18.2 | dre:368620/dre:570453/dre:406803/dre:406803/dre:571189/dre:570148/dre:735310/dre:556826 | 8 |
| dre00360 | Phenylalanine metabolism | 4/511 | 22/8379 | 0.0416425 | 0.4496866 | 541429/561410\_1/445287/394142 | aoc2/tat/hpdb/hpda | dre:541429/dre:561410/dre:445287/dre:394142 | 4 |
| dre00450 | Selenocompound metabolism | 4/511 | 22/8379 | 0.0416425 | 0.4496866 | 103911233/101884223/445818/103911241 | LOC103911233/LOC101884223/cthl/LOC103911241 | dre:103911233/dre:101884223/dre:445818/dre:571201 | 4 |
| dre00500 | Starch and sucrose metabolism | 6/511 | 44/8379 | 0.0491876 | 0.4496866 | 393444/406539/445049/100333665/321224/751668 | pygmb/amy2a/zgc:92137/gbe1a/hkdc1/gck | dre:393444/dre:406539/dre:445049/dre:100333665/dre:321224/dre:751668 | 6 |
| dre00130 | Ubiquinone and other terpenoid-quinone biosynthesis | 3/511 | 14/8379 | 0.0496150 | 0.4496866 | 561410\_1/445287/394142 | tat/hpdb/hpda | dre:561410/dre:445287/dre:394142 | 3 |
| dre00260 | Glycine, serine and threonine metabolism | 7/511 | 56/8379 | 0.0524806 | 0.4496866 | 541429/541329/445818/394103/405800/619259/563642 | aoc2/gcsha/cthl/sardh/dao.2/dao.1/sdsl | dre:541429/dre:541329/dre:445818/dre:394103/dre:405800/dre:619259/dre:563642 | 7 |
| dre00620 | Pyruvate metabolism | 8/511 | 68/8379 | 0.0537296 | 0.4496866 | 335715/116517/445233/406473/368239/559403\_1/406702/445326\_1 | mdh1ab/adh5/me2/pck2/aldh2.2/acaca/pdha1a/akr1a1a | dre:335715/dre:116517/dre:445233/dre:406473/dre:368239/dre:559403/dre:406702/dre:445326 | 8 |
| dre00030 | Pentose phosphate pathway | 6/511 | 45/8379 | 0.0539636 | 0.4496866 | 560827\_1/406762\_1/403070\_1/570579\_1/561416\_1/568001\_3 | prps1b/pgd/rgn/g6pd/pfkpb/pfkmb | dre:560827/dre:406762/dre:403070/dre:570579/dre:561416/dre:568001 | 6 |
| dre00100 | Steroid biosynthesis | 4/511 | 24/8379 | 0.0550049 | 0.4496866 | 322481/100004700/565117/406662\_2 | cel.1/cyp24a1/cel.2/msmo1 | dre:322481/dre:100004700/dre:565117/dre:406662 | 4 |
| dre04070 | Phosphatidylinositol signaling system | 15/511 | 157/8379 | 0.0554688 | 0.4496866 | 100002015\_1/336121/724010\_1/368717/337236\_2/559938\_1/100000252\_3/100006675/337236/566642\_1/571356/678558\_1/571356\_2/100000252/559198 | cds1/calm2a/dgkaa/itpka/synj1/ppip5k1b/pik3r3b/prkcba/synj1/tmem55a/pik3c2a/mtmr7a/pik3c2a/pik3r3b/mtmr7b | dre:100002015/dre:192322/dre:724010/dre:368717/dre:337236/dre:559938/dre:100000252/dre:100006675/dre:337236/dre:566642/dre:571356/dre:678558/dre:571356/dre:100000252/dre:559198 | 15 |
| dre01232 | Nucleotide metabolism | 13/511 | 131/8379 | 0.0558434 | 0.4496866 | 100330864/334431\_1/492517/550560\_1/100005763/103910413\_1/393744\_1/553639\_1/402953\_1/333997/558466\_1/447905/333997\_1 | LOC100330864/adsl/zgc:103586/entpd6/uck2a/tymp/dctpp1/gmpr/pnp6/ampd3b/uckl1b/entpd2a.1/ampd3b | dre:100330864/dre:334431/dre:492517/dre:550560/dre:100005763/dre:103910413/dre:393744/dre:553639/dre:402953/dre:333997/dre:558466/dre:447905/dre:333997 | 13 |
| dre00020 | Citrate cycle (TCA cycle) | 6/511 | 47/8379 | 0.0643307 | 0.4921300 | 335715/406473/100006589\_1/550579\_1/317746/406702 | mdh1ab/pck2/idh1/idh3g/suclg2/pdha1a | dre:335715/dre:406473/dre:100006589/dre:550579/dre:317746/dre:406702 | 6 |
| dre00900 | Terpenoid backbone biosynthesis | 4/511 | 26/8379 | 0.0704047 | 0.5129488 | 334380\_1/559054\_1/557720\_1/100003383 | zmpste24/hmgcra/rce1a/pmvk | dre:334380/dre:559054/dre:557720/dre:100003383 | 4 |
| dre00350 | Tyrosine metabolism | 6/511 | 50/8379 | 0.0819216 | 0.5697272 | 116517/541429/561410\_1/445287/394142/30505 | adh5/aoc2/tat/hpdb/hpda/dbh | dre:116517/dre:541429/dre:561410/dre:445287/dre:394142/dre:30505 | 6 |
| dre03460 | Fanconi anemia pathway | 7/511 | 63/8379 | 0.0875491 | 0.5823916 | 100538153/558923/406487/794038/402936\_1/450081/570043\_1 | slx4/palb2/rad51/brip1/fancg/rad51c/rev1 | dre:100538153/dre:558923/dre:406487/dre:794038/dre:402936/dre:450081/dre:570043 | 7 |
| dre00010 | Glycolysis / Gluconeogenesis | 10/511 | 103/8379 | 0.0966017 | 0.6158357 | 560753\_1/116517/406473/368239/561416\_1/406702/445326\_1/321224/751668/568001\_3 | tpi1b/adh5/pck2/aldh2.2/pfkpb/pdha1a/akr1a1a/hkdc1/gck/pfkmb | dre:560753/dre:116517/dre:406473/dre:368239/dre:561416/dre:406702/dre:445326/dre:321224/dre:751668/dre:568001 | 10 |
| dre03430 | Mismatch repair | 4/511 | 30/8379 | 0.1069861 | 0.6503796 | 556995\_1/565400/568323\_1/565400\_1 | lig1/msh3/mlh3/msh3 | dre:556995/dre:565400/dre:568323/dre:565400 | 4 |
| dre00380 | Tryptophan metabolism | 7/511 | 67/8379 | 0.1123625 | 0.6503796 | 103911233/447801\_1/368239/393860/571967/555401/103911241 | LOC103911233/aldh8a1/aldh2.2/gcdha/ido1/aoc1/LOC103911241 | dre:103911233/dre:447801/dre:368239/dre:393860/dre:571967/dre:555401/dre:571201 | 7 |
| dre00052 | Galactose metabolism | 5/511 | 43/8379 | 0.1189190 | 0.6503796 | 561416\_1/450083/321224/751668/568001\_3 | pfkpb/gla/hkdc1/gck/pfkmb | dre:561416/dre:450083/dre:321224/dre:751668/dre:568001 | 5 |
| dre00603 | Glycosphingolipid biosynthesis - globo and isoglobo series | 3/511 | 20/8379 | 0.1190237 | 0.6503796 | 447881/450083/100005375 | gbgt1l3/gla/si:ch211-135f11.4 | dre:447881/dre:450083/dre:100005375 | 3 |
| dre01230 | Biosynthesis of amino acids | 10/511 | 109/8379 | 0.1274260 | 0.6722822 | 334195\_1/560753\_1/560827\_1/100006589\_1/550579\_1/393799/561416\_1/445818/563642/568001\_3 | mat1a/tpi1b/prps1b/idh1/idh3g/pycr1a/pfkpb/cthl/sdsl/pfkmb | dre:334195/dre:560753/dre:560827/dre:100006589/dre:550579/dre:393799/dre:561416/dre:445818/dre:563642/dre:568001 | 10 |
| dre00591 | Linoleic acid metabolism | 4/511 | 33/8379 | 0.1389784 | 0.7087898 | 406370/393592/553969/560649 | pla2g6/cyp2p9/cyp3a65/si:ch211-214p16.3 | dre:406370/dre:393592/dre:553969/dre:560649 | 4 |
| dre00480 | Glutathione metabolism | 8/511 | 85/8379 | 0.1456970 | 0.7190852 | 100330864/406762\_1/570579\_1/100006589\_1/555317/393493/431762\_1/450084\_1 | LOC100330864/pgd/g6pd/idh1/si:ch211-276a23.5/gstt2/mgst1.2/gss | dre:100330864/dre:406762/dre:570579/dre:100006589/dre:555317/dre:393493/dre:431762/dre:450084 | 8 |
| dre00561 | Glycerolipid metabolism | 8/511 | 89/8379 | 0.1745226 | 0.8344360 | 724010\_1/322481/368239/799099\_1/565117/450083/445326\_1/572841 | dgkaa/cel.1/aldh2.2/lpin1/cel.2/gla/akr1a1a/mboat1 | dre:724010/dre:322481/dre:368239/dre:799099/dre:565117/dre:450083/dre:445326/dre:572841 | 8 |
| dre03266 | Virion - Herpesvirus | 2/511 | 13/8379 | 0.1860236 | 0.8624729 | 791704/564266 | zgc:153759/itgb3a | dre:791704/dre:564266 | 2 |
| dre00790 | Folate biosynthesis | 4/511 | 38/8379 | 0.1993219 | 0.8969487 | 557552/791448\_1/100330410/574009 | zgc:153031/qdprb1/alp3/alpi.2 | dre:557552/dre:791448/dre:100330410/dre:574009 | 4 |
| dre00592 | alpha-Linolenic acid metabolism | 3/511 | 26/8379 | 0.2092239 | 0.9146072 | 406370/140615/560649 | pla2g6/fads2/si:ch211-214p16.3 | dre:406370/dre:140615/dre:560649 | 3 |
| dre00565 | Ether lipid metabolism | 6/511 | 67/8379 | 0.2231073 | 0.9235145 | 406370/492735/565743\_1/560225/560649/567170\_1 | pla2g6/si:ch211-212o1.2/pld2/cept1a/si:ch211-214p16.3/pafah1b2 | dre:406370/dre:492735/dre:565743/dre:560225/dre:560649/dre:567170 | 6 |
| dre00330 | Arginine and proline metabolism | 7/511 | 81/8379 | 0.2233336 | 0.9235145 | 321892\_1/368239/393799/405800/555401/619259/327506\_1 | ckmt1/aldh2.2/pycr1a/dao.2/aoc1/dao.1/ckmt2a | dre:321892/dre:368239/dre:393799/dre:405800/dre:555401/dre:619259/dre:327506 | 7 |
| dre00051 | Fructose and mannose metabolism | 5/511 | 54/8379 | 0.2315364 | 0.9322385 | 560753\_1/561416\_1/436909/321224/568001\_3 | tpi1b/pfkpb/khk/hkdc1/pfkmb | dre:560753/dre:561416/dre:436909/dre:321224/dre:568001 | 5 |
| dre04210 | Apoptosis | 17/511 | 232/8379 | 0.2494078 | 0.9601306 | 57934\_1/431763/556410\_1/100000252\_3/394198/570238/567021/569298/101886621\_1/768248/561021/552927/58133/431777/791531/100000252/100135062\_2 | actb1/gadd45ab/atf4b/pik3r3b/fosab/fosaa/ern2/ctsbb/LOC101886621/fas/si:dkey-103e21.5/casp6/ngfb/tuba7l/casp6l1/pik3r3b/csf2rb | dre:57934/dre:431763/dre:556410/dre:100000252/dre:394198/dre:570238/dre:567021/dre:569298/dre:101886621/dre:768248/dre:561021/dre:552927/dre:58133/dre:431777/dre:791531/dre:100000252/dre:100135062 | 17 |
| dre00240 | Pyrimidine metabolism | 7/511 | 84/8379 | 0.2510145 | 0.9601306 | 100330864/492517/550560\_1/100005763/103910413\_1/393744\_1/558466\_1 | LOC100330864/zgc:103586/entpd6/uck2a/tymp/dctpp1/uckl1b | dre:100330864/dre:492517/dre:550560/dre:100005763/dre:103910413/dre:393744/dre:558466 | 7 |
| dre00512 | Mucin type O-glycan biosynthesis | 4/511 | 43/8379 | 0.2659074 | 0.9630317 | 324510\_1/557675\_1/555344/337131 | gcnt4a/c1galt1a/si:dkey-202e17.1/c1galt1b | dre:324510/dre:557675/dre:555344/dre:337131 | 4 |
| dre04150 | mTOR signaling pathway | 18/511 | 251/8379 | 0.2705028 | 0.9630317 | 324140\_1/796322/436718/79380/799099\_1/359840/100000252\_3/100006675/553214\_1/100330617\_1/555584\_1/100000252/359839/245702/100009657\_1/556087/58072\_1/573492\_1 | sgk1/slc3a2a/rhocb/eif4ea/lpin1/atp6v1b2/pik3r3b/prkcba/eif4ba/tsc1b/prr5a/pik3r3b/atp6v1ba/igf1rb/rps6ka3b/wnt2bb/fzd8b/rragca | dre:324140/dre:796322/dre:436718/dre:79380/dre:799099/dre:359840/dre:100000252/dre:100006675/dre:553214/dre:100330617/dre:555584/dre:100000252/dre:359839/dre:245702/dre:100009657/dre:556087/dre:58072/dre:573492 | 18 |
| dre04137 | Mitophagy - animal | 9/511 | 116/8379 | 0.2757796 | 0.9630317 | 550134\_1/novel.905/556410\_1/555713/326974\_1/564996/492676/561933\_2/561933\_1 | ubb/-/atf4b/nbr1b/gabarapa/tfe3b/tbc1d15/rhot1b/rhot1b | dre:550134/dre:777766/dre:556410/dre:555713/dre:326974/dre:564996/dre:492676/dre:561933/dre:561933 | 9 |
| dre04914 | Progesterone-mediated oocyte maturation | 10/511 | 133/8379 | 0.2926549 | 0.9630317 | 323509\_1/562552/767751\_1/100000252\_3/568726/100000252/560807/245702/100009657\_1/280649\_1 | gnaia/zgc:171775/ccnb3/pik3r3b/adcy7/pik3r3b/adcy6b/igf1rb/rps6ka3b/plk1 | dre:323509/dre:562552/dre:767751/dre:100000252/dre:568726/dre:100000252/dre:560807/dre:245702/dre:100009657/dre:280649 | 10 |
| dre04068 | FoxO signaling pathway | 15/511 | 210/8379 | 0.2998096 | 0.9630317 | 324140\_1/406473/117508\_1/431763/566841/562552/117508/767751\_1/100000252\_3/326974\_1/565576/100000252/245702/280649\_1/559723 | sgk1/pck2/klf2a/gadd45ab/crebbpa/zgc:171775/klf2a/ccnb3/pik3r3b/gabarapa/plk2b/pik3r3b/igf1rb/plk1/tgfb2l | dre:324140/dre:406473/dre:117508/dre:431763/dre:566841/dre:562552/dre:117508/dre:767751/dre:100000252/dre:326974/dre:565576/dre:100000252/dre:245702/dre:280649/dre:559723 | 15 |
| dre04370 | VEGF signaling pathway | 8/511 | 104/8379 | 0.3006202 | 0.9630317 | 559020/562552/793158\_1/100000252\_3/100006675/556226\_1/565241/100000252 | ptgs2b/zgc:171775/cdc42l/pik3r3b/prkcba/cdc42l2/ppp3ccb/pik3r3b | dre:559020/dre:562552/dre:793158/dre:100000252/dre:100006675/dre:556226/dre:565241/dre:100000252 | 8 |
| dre00730 | Thiamine metabolism | 2/511 | 18/8379 | 0.3011933 | 0.9630317 | 100330410/574009 | alp3/alpi.2 | dre:100330410/dre:574009 | 2 |
| dre04060 | Cytokine-cytokine receptor interaction | 17/511 | 242/8379 | 0.3072566 | 0.9630317 | 799354/368326\_1/58151\_1/561050\_1/799279/368438\_1/562247/768248/100135755/58133/791704/100006078/100135062\_2/100001424/100333914/30125/559723 | cxcl12b/il13ra2/cxcl14/ackr3b/LOC799279/cntfr/clcf1/fas/il6r/ngfb/zgc:153759/ccr9b/csf2rb/ghra/ccl25a/gdf3/tgfb2l | dre:799354/dre:368326/dre:58151/dre:561050/dre:799279/dre:368438/dre:562247/dre:768248/dre:100135755/dre:58133/dre:791704/dre:100006078/dre:100135062/dre:100001424/dre:100333914/dre:30125/dre:559723 | 17 |
| dre03320 | PPAR signaling pathway | 8/511 | 106/8379 | 0.3189373 | 0.9630317 | 550134\_1/406473/novel.905/334529/30355\_1/553512/140615/550432 | ubb/pck2/-/aqp7/apoa1a/slc27a1b/fads2/slc27a4 | dre:550134/dre:406473/dre:777766/dre:334529/dre:30355/dre:553512/dre:140615/dre:550432 | 8 |
| dre04142 | Lysosome | 15/511 | 214/8379 | 0.3255492 | 0.9630317 | 565099/100003043\_1/569298/450083/368911/436725\_1/553423/566517/445244/559575/327237\_1/570148\_1/406511\_1/393657/567720 | zgc:193725/lamp3/ctsbb/gla/laptm4b/acp5b/sumf1/aga/ap1m3/si:ch211-15d5.11/ap1s2/abcb9/sort1a/zgc:66024/idua | dre:565099/dre:100003043/dre:569298/dre:450083/dre:368911/dre:436725/dre:553423/dre:566517/dre:445244/dre:559575/dre:327237/dre:570148/dre:406511/dre:393657/dre:567720 | 15 |
| dre00562 | Inositol phosphate metabolism | 9/511 | 123/8379 | 0.3353784 | 0.9630317 | 560753\_1/368717/337236\_2/337236/100006223/571356/678558\_1/571356\_2/559198 | tpi1b/itpka/synj1/synj1/si:dkey-266f7.9/pik3c2a/mtmr7a/pik3c2a/mtmr7b | dre:560753/dre:368717/dre:337236/dre:337236/dre:100006223/dre:571356/dre:678558/dre:571356/dre:559198 | 9 |
| dre04810 | Regulation of actin cytoskeleton | 27/511 | 404/8379 | 0.3371231 | 0.9630317 | 57934\_1/799354/570868\_1/286739/436718/566764\_1/793158\_1/100000252\_3/100331581/100002872\_2/677750/556226\_1/796490\_2/494456/100005469\_1/570832/553493\_1/30429/58075/100000252/794365/103909601/564565/798694\_1/570832\_1/564266/101882911 | actb1/cxcl12b/itga1/msna/rhocb/scin/cdc42l/pik3r3b/si:dkey-44g17.6/cyfip2/myl2b/cdc42l2/pdgfbb/fgf23/fn1a/c7b/arhgef7a/mylpfa/fgd/pik3r3b/si:dkey-163m14.7/LOC103909601/arhgap35/LOC798694/c7b/itgb3a/LOC101882911 | dre:57934/dre:799354/dre:570868/dre:286739/dre:436718/dre:566764/dre:793158/dre:100000252/dre:100331581/dre:100002872/dre:677750/dre:556226/dre:796490/dre:494456/dre:100005469/dre:570832/dre:553493/dre:30429/dre:58075/dre:100000252/dre:794365/dre:103909601/dre:564565/dre:798694/dre:570832/dre:564266/dre:321070 | 27 |
| dre04330 | Notch signaling pathway | 7/511 | 93/8379 | 0.3390392 | 0.9630317 | 678649\_1/566841/393500\_1/557340\_1/30120\_1/497616/140422 | zgc:136929/crebbpa/zgc:66440/atxn1a/dlc/numbl/jag2b | dre:678649/dre:566841/dre:393500/dre:557340/dre:30120/dre:497616/dre:140422 | 7 |
| dre05132 | Salmonella infection | 27/511 | 406/8379 | 0.3467975 | 0.9630317 | 57934\_1/386590\_1/562529\_1/100004870\_1/562372\_1/558917\_1/562552/436718/563023\_1/793158\_1/394198/570238/798985\_1/100002872\_2/101882034/677750/566185/393653\_1/556226\_1/571356/561021/571356\_2/110437957/548603\_1/431777/30429/101882911 | actb1/hsp90b1/flna/exoc2/snx18a/snx9b/zgc:171775/rhocb/flnb/cdc42l/fosab/fosaa/vps16/cyfip2/LOC101882034/myl2b/caspbl/rhogd/cdc42l2/pik3c2a/si:dkey-103e21.5/pik3c2a/LOC110437957/snx18b/tuba7l/mylpfa/LOC101882911 | dre:57934/dre:386590/dre:562529/dre:100004870/dre:562372/dre:558917/dre:562552/dre:436718/dre:563023/dre:793158/dre:394198/dre:570238/dre:798985/dre:100002872/dre:406297/dre:677750/dre:566185/dre:393653/dre:556226/dre:571356/dre:561021/dre:571356/dre:373874/dre:548603/dre:431777/dre:30429/dre:321070 | 27 |
| dre04625 | C-type lectin receptor signaling pathway | 11/511 | 156/8379 | 0.3537506 | 0.9630317 | 336121/559020/562552/436718/100000252\_3/368241/566185/556277/561021/565241/100000252 | calm2a/ptgs2b/zgc:171775/rhocb/pik3r3b/egr2a/caspbl/cblb/si:dkey-103e21.5/ppp3ccb/pik3r3b | dre:192322/dre:559020/dre:562552/dre:436718/dre:100000252/dre:368241/dre:566185/dre:556277/dre:561021/dre:565241/dre:100000252 | 11 |
| dre01240 | Biosynthesis of cofactors | 16/511 | 234/8379 | 0.3541004 | 0.9630317 | 334195\_1/334431\_1/557552/403070\_1/368239/445287/394142/571967/445083/393580/406789/445326\_1/110438097/100330410/574009/450084\_1 | mat1a/adsl/zgc:153031/rgn/aldh2.2/hpdb/hpda/ido1/dhrs3a/bco1l/pank1a/akr1a1a/LOC110438097/alp3/alpi.2/gss | dre:334195/dre:334431/dre:557552/dre:403070/dre:368239/dre:445287/dre:394142/dre:571967/dre:445083/dre:393580/dre:406789/dre:445326/dre:572070/dre:100330410/dre:574009/dre:450084 | 16 |
| dre03013 | Nucleocytoplasmic transport | 9/511 | 129/8379 | 0.3880595 | 0.9630317 | 393693\_1/100005893/393973/324342\_1/406860/321744\_1/100004503/407710/333980 | sap18/magoh/rae1/xpo4/ipo9/nup88/eef1a1b/xpo7/xpo6 | dre:393693/dre:100005893/dre:393973/dre:324342/dre:406860/dre:321744/dre:100004503/dre:407710/dre:333980 | 9 |
| dre04910 | Insulin signaling pathway | 14/511 | 212/8379 | 0.4186645 | 0.9630317 | 100001260\_1/393444/406473/336121/79380/100000252\_3/556277/100330617\_1/559403\_1/100000252/321224/751668/406459/569706\_1 | ppp1r3ab/pygmb/pck2/calm2a/eif4ea/pik3r3b/cblb/tsc1b/acaca/pik3r3b/hkdc1/gck/zgc:77112/phkb | dre:100001260/dre:393444/dre:406473/dre:192322/dre:79380/dre:100000252/dre:556277/dre:100330617/dre:559403/dre:100000252/dre:321224/dre:751668/dre:406459/dre:569706 | 14 |
| dre01040 | Biosynthesis of unsaturated fatty acids | 3/511 | 39/8379 | 0.4279441 | 0.9630317 | 140615/393441/554145 | fads2/hacd3/elovl8b | dre:140615/dre:393441/dre:554145 | 3 |
| dre00310 | Lysine degradation | 7/511 | 102/8379 | 0.4303127 | 0.9630317 | 368239/393860/556077\_1/100003615/572649/402830/556535 | aldh2.2/gcdha/plod3/prdm2a/bbox1/ehmt1b/setd1a | dre:368239/dre:393860/dre:556077/dre:100003615/dre:572649/dre:402830/dre:556535 | 7 |
| dre03015 | mRNA surveillance pathway | 8/511 | 118/8379 | 0.4315427 | 0.9630317 | 386630\_1/324267\_1/393693\_1/100005893/100002680\_2/321035\_1/559392\_1/562282 | tardbpl/hbs1l/sap18/magoh/msi2a/pabpc4/ppp2r2cb/ppp2r5b | dre:386630/dre:324267/dre:393693/dre:100005893/dre:100002680/dre:321035/dre:559392/dre:562282 | 8 |
| dre00910 | Nitrogen metabolism | 2/511 | 24/8379 | 0.4350650 | 0.9630317 | 569989\_1/767680 | ca5a/zgc:153760 | dre:569989/dre:767680 | 2 |
| dre00280 | Valine, leucine and isoleucine degradation | 5/511 | 71/8379 | 0.4375541 | 0.9630317 | 368239/437019\_1/394130\_1/797311/777632 | aldh2.2/pcca/acad8/LOC797311/mccc1 | dre:368239/dre:437019/dre:394130/dre:797311/dre:777632 | 5 |
| dre00564 | Glycerophospholipid metabolism | 9/511 | 135/8379 | 0.4411421 | 0.9630317 | 100002015\_1/724010\_1/406370/799099\_1/334017\_1/565743\_1/560225/560649/572841 | cds1/dgkaa/pla2g6/lpin1/pla1a/pld2/cept1a/si:ch211-214p16.3/mboat1 | dre:100002015/dre:724010/dre:406370/dre:799099/dre:334017/dre:565743/dre:560225/dre:560649/dre:572841 | 9 |
| dre03060 | Protein export | 2/511 | 25/8379 | 0.4560962 | 0.9630317 | 436794/445299\_1 | sec11a/immp2l | dre:436794/dre:445299 | 2 |
| dre00640 | Propanoate metabolism | 3/511 | 41/8379 | 0.4606045 | 0.9630317 | 437019\_1/317746/559403\_1 | pcca/suclg2/acaca | dre:437019/dre:317746/dre:559403 | 3 |
| dre00400 | Phenylalanine, tyrosine and tryptophan biosynthesis | 1/511 | 10/8379 | 0.4671929 | 0.9630317 | 561410\_1 | tat | dre:561410 | 1 |
| dre05168 | Herpes simplex virus 1 infection | 14/511 | 220/8379 | 0.4746380 | 0.9630317 | 100149189/100000252\_3/30762/58113/368878/100330617\_1/768248/561021/30763/791704/100000252/571282/564266/556826\_1 | si:ch73-237c6.1/pik3r3b/mhc2dab/cd74a/si:busm1-266f07.2/tsc1b/fas/si:dkey-103e21.5/mhc2dcb/zgc:153759/pik3r3b/LOC571282/itgb3a/si:dkey-57h18.2 | dre:100149189/dre:100000252/dre:30762/dre:58113/dre:368878/dre:100330617/dre:768248/dre:561021/dre:30763/dre:791704/dre:100000252/dre:571282/dre:564266/dre:556826 | 14 |
| dre00650 | Butanoate metabolism | 2/511 | 26/8379 | 0.4766689 | 0.9630317 | 797311/565235\_1 | LOC797311/aldh5a1 | dre:797311/dre:565235 | 2 |
| dre04912 | GnRH signaling pathway | 10/511 | 158/8379 | 0.4990611 | 0.9630317 | 336121/30498\_1/562552/556410\_1/793158\_1/100006675/568726/556226\_1/565743\_1/560807 | calm2a/egr1/zgc:171775/atf4b/cdc42l/prkcba/adcy7/cdc42l2/pld2/adcy6b | dre:192322/dre:30498/dre:562552/dre:556410/dre:793158/dre:100006675/dre:568726/dre:556226/dre:565743/dre:560807 | 10 |
| dre00740 | Riboflavin metabolism | 1/511 | 11/8379 | 0.4997254 | 0.9630317 | 436725\_1 | acp5b | dre:436725 | 1 |
| dre00230 | Purine metabolism | 12/511 | 192/8379 | 0.5082990 | 0.9630317 | 100330864/334431\_1/560827\_1/568726/550560\_1/553639\_1/402953\_1/333997/560807/447905/140425/333997\_1 | LOC100330864/adsl/prps1b/adcy7/entpd6/gmpr/pnp6/ampd3b/adcy6b/entpd2a.1/gc2/ampd3b | dre:100330864/dre:334431/dre:560827/dre:568726/dre:550560/dre:553639/dre:402953/dre:333997/dre:560807/dre:447905/dre:140425/dre:333997 | 12 |
| dre04145 | Phagosome | 13/511 | 209/8379 | 0.5123510 | 0.9630317 | 57934\_1/359840/100004793/30762/368878/100537272/403014/30763/431777/359839/571282/564266/556826\_1 | actb1/atp6v1b2/mrc2/mhc2dab/si:busm1-266f07.2/LOC100537272/ncf4/mhc2dcb/tuba7l/atp6v1ba/LOC571282/itgb3a/si:dkey-57h18.2 | dre:57934/dre:359840/dre:100004793/dre:30762/dre:368878/dre:100537272/dre:403014/dre:30763/dre:431777/dre:359839/dre:571282/dre:564266/dre:556826 | 13 |
| dre04270 | Vascular smooth muscle contraction | 14/511 | 226/8379 | 0.5161124 | 0.9630317 | 337226\_1/336121/436718/798632/406370/100006675/436966\_2/568726/403114\_1/567131/393783\_1/560807/492486/101882911 | zgc:153867/calm2a/rhocb/myl6/pla2g6/prkcba/zgc:91818/adcy7/adm2a/calcrlb/ppp1r14ab/adcy6b/adra1bb/LOC101882911 | dre:337226/dre:192322/dre:436718/dre:798632/dre:406370/dre:100006675/dre:436966/dre:568726/dre:403114/dre:567131/dre:393783/dre:560807/dre:492486/dre:321070 | 14 |
| dre00770 | Pantothenate and CoA biosynthesis | 2/511 | 28/8379 | 0.5163537 | 0.9630317 | 368239/406789 | aldh2.2/pank1a | dre:368239/dre:406789 | 2 |
| dre04520 | Adherens junction | 12/511 | 194/8379 | 0.5230925 | 0.9630317 | 57934\_1/566841/436718/793158\_1/677750/335573\_1/556226\_1/393527/30429/245702/564805\_1/101882911 | actb1/crebbpa/rhocb/cdc42l/myl2b/ptpn6/cdc42l2/pdcd10a/mylpfa/igf1rb/arhgap29a/LOC101882911 | dre:57934/dre:566841/dre:436718/dre:793158/dre:677750/dre:335573/dre:556226/dre:393527/dre:30429/dre:245702/dre:378998/dre:321070 | 12 |
| dre04512 | ECM-receptor interaction | 8/511 | 128/8379 | 0.5241477 | 0.9630317 | 570868\_1/563428\_1/321243\_1/30037/798998/322012\_1/100005469\_1/564266 | itga1/fras1/lama5/tnc/gp1bb/hmmr/fn1a/itgb3a | dre:570868/dre:563428/dre:321243/dre:30037/dre:798998/dre:322012/dre:100005469/dre:564266 | 8 |
| dre00920 | Sulfur metabolism | 1/511 | 12/8379 | 0.5302751 | 0.9630317 | 393542\_1 | selenbp1 | dre:393542 | 1 |
| dre00062 | Fatty acid elongation | 3/511 | 46/8379 | 0.5383633 | 0.9630317 | 393441/554145/393657 | hacd3/elovl8b/zgc:66024 | dre:393441/dre:554145/dre:393657 | 3 |
| dre00630 | Glyoxylate and dicarboxylate metabolism | 3/511 | 46/8379 | 0.5383633 | 0.9630317 | 335715/437019\_1/541329 | mdh1ab/pcca/gcsha | dre:335715/dre:437019/dre:541329 | 3 |
| dre04146 | Peroxisome | 7/511 | 114/8379 | 0.5486265 | 0.9630317 | 100093708\_1/100006589\_1/405800/406803\_1/406803\_2/100003383/619259 | slc25a17/idh1/dao.2/abcd3a/abcd3a/pmvk/dao.1 | dre:100093708/dre:100006589/dre:405800/dre:406803/dre:406803/dre:100003383/dre:619259 | 7 |
| dre00340 | Histidine metabolism | 2/511 | 30/8379 | 0.5539911 | 0.9630317 | 368239/555401 | aldh2.2/aoc1 | dre:368239/dre:555401 | 2 |
| dre01210 | 2-Oxocarboxylic acid metabolism | 2/511 | 30/8379 | 0.5539911 | 0.9630317 | 100006589\_1/550579\_1 | idh1/idh3g | dre:100006589/dre:550579 | 2 |
| dre04120 | Ubiquitin mediated proteolysis | 12/511 | 199/8379 | 0.5594552 | 0.9630317 | 192341/550134\_1/692325/564149\_1/novel.905/323951\_2/556277/564866\_1/559439\_1/393445\_1/559981/100151173\_1 | tceb2/ubb/flj11011l/pias2/-/klhl13/cblb/trip12/huwe1/ube3c/si:ch73-190m4.1/si:rp71-68n21.9 | dre:192341/dre:550134/dre:692325/dre:564149/dre:777766/dre:323951/dre:556277/dre:564866/dre:559439/dre:393445/dre:559981/dre:100151173 | 12 |
| dre00514 | Other types of O-glycan biosynthesis | 4/511 | 65/8379 | 0.5661904 | 0.9630317 | 556077\_1/557675\_1/555344/337131 | plod3/c1galt1a/si:dkey-202e17.1/c1galt1b | dre:556077/dre:557675/dre:555344/dre:337131 | 4 |
| dre03050 | Proteasome | 4/511 | 65/8379 | 0.5661904 | 0.9630317 | 30387/796450\_1/326884\_1/436816 | psmb5/adrm1/psmc4/zgc:92791 | dre:30387/dre:796450/dre:326884/dre:436816 | 4 |
| dre03030 | DNA replication | 3/511 | 48/8379 | 0.5676251 | 0.9630317 | 393241\_1/393774/556995\_1 | rnaseh2b/pole3/lig1 | dre:393241/dre:393774/dre:556995 | 3 |
| dre00532 | Glycosaminoglycan biosynthesis - chondroitin sulfate / dermatan sulfate | 2/511 | 31/8379 | 0.5720177 | 0.9630317 | 100331876/100538167 | LOC100331876/si:dkey-26i13.6 | dre:100331876/dre:100538167 | 2 |
| dre03008 | Ribosome biogenesis in eukaryotes | 5/511 | 83/8379 | 0.5769851 | 0.9630317 | 393107\_2/407084\_1/393107\_1/492761\_1/553787\_1 | utp6/riok2/utp6/tbl3/pop4 | dre:393107/dre:407084/dre:393107/dre:492761/dre:553787 | 5 |
| dre04510 | Focal adhesion | 21/511 | 354/8379 | 0.5863308 | 0.9630317 | 57934\_1/562529\_1/570868\_1/436718/321243\_1/563023\_1/793158\_1/100000252\_3/100006675/30037/677750/556226\_1/796490\_2/100005469\_1/30429/100000252/245702/103909601/564565/564266/101882911 | actb1/flna/itga1/rhocb/lama5/flnb/cdc42l/pik3r3b/prkcba/tnc/myl2b/cdc42l2/pdgfbb/fn1a/mylpfa/pik3r3b/igf1rb/LOC103909601/arhgap35/itgb3a/LOC101882911 | dre:57934/dre:562529/dre:570868/dre:436718/dre:321243/dre:563023/dre:793158/dre:100000252/dre:100006675/dre:30037/dre:677750/dre:556226/dre:796490/dre:100005469/dre:30429/dre:100000252/dre:245702/dre:103909601/dre:564565/dre:564266/dre:321070 | 21 |
| dre04115 | p53 signaling pathway | 6/511 | 101/8379 | 0.5863903 | 0.9630317 | 100330864/431763/171473/436699\_1/768248/561021 | LOC100330864/gadd45ab/ccng1/zgc:92658/fas/si:dkey-103e21.5 | dre:100330864/dre:431763/dre:171473/dre:436699/dre:768248/dre:561021 | 6 |
| dre04514 | Cell adhesion molecules | 12/511 | 204/8379 | 0.5947310 | 0.9630317 | 30194/30762/793143/30634\_1/368878/797651\_1/30763/571282/541474/100151365\_1/100005566/562934\_1 | alcama/mhc2dab/si:dkey-98f17.3/nadl1.2/si:busm1-266f07.2/jam3a/mhc2dcb/LOC571282/cntn1b/si:dkey-1h24.6/f11r.2/esamb | dre:30194/dre:30762/dre:793143/dre:30634/dre:368878/dre:797651/dre:30763/dre:571282/dre:541474/dre:100151365/dre:100005566/dre:562934 | 12 |
| dre04916 | Melanogenesis | 10/511 | 171/8379 | 0.6015211 | 0.9630317 | 323509\_1/336121/566841/100006675/568726/560807/100538072/556087/553181\_1/58072\_1 | gnaia/calm2a/crebbpa/prkcba/adcy7/adcy6b/creb3l3b/wnt2bb/kitlgb/fzd8b | dre:323509/dre:192322/dre:566841/dre:100006675/dre:568726/dre:560807/dre:100538072/dre:556087/dre:553181/dre:58072 | 10 |
| dre01250 | Biosynthesis of nucleotide sugars | 3/511 | 51/8379 | 0.6093148 | 0.9630317 | 100885772/321224/751668 | cmasb/hkdc1/gck | dre:100885772/dre:321224/dre:751668 | 3 |
| dre04140 | Autophagy - animal | 14/511 | 241/8379 | 0.6150897 | 0.9630317 | 110438871/792229\_1/449923/100000252\_3/326974\_1/567021/449923\_1/569298/100330617\_1/563140/406843\_1/100000252/245702/573492\_1 | LOC110438871/sh3glb1a/si:ch73-234b20.5/pik3r3b/gabarapa/ern2/si:ch73-234b20.5/ctsbb/tsc1b/si:ch211-11n16.2/igbp1/pik3r3b/igf1rb/rragca | dre:110438871/dre:792229/dre:449923/dre:100000252/dre:326974/dre:567021/dre:449923/dre:569298/dre:100330617/dre:563140/dre:406843/dre:100000252/dre:245702/dre:573492 | 14 |
| dre00760 | Nicotinate and nicotinamide metabolism | 3/511 | 52/8379 | 0.6226010 | 0.9630317 | 402953\_1/101886891/110438097 | pnp6/LOC101886891/LOC110438097 | dre:402953/dre:101886891/dre:572070 | 3 |
| dre03450 | Non-homologous end-joining | 1/511 | 16/8379 | 0.6349519 | 0.9630317 | 566285\_1 | dclre1c | dre:566285 | 1 |
| dre00983 | Drug metabolism - other enzymes | 7/511 | 124/8379 | 0.6385198 | 0.9630317 | 100330864/492517/100005763/103910413\_1/393493/558466\_1/431762\_1 | LOC100330864/zgc:103586/uck2a/tymp/gstt2/uckl1b/mgst1.2 | dre:100330864/dre:492517/dre:100005763/dre:103910413/dre:393493/dre:558466/dre:431762 | 7 |
| dre04623 | Cytosolic DNA-sensing pathway | 4/511 | 72/8379 | 0.6476738 | 0.9630317 | 562329/566185/394162\_1/561021 | polr3b/caspbl/polr3f/si:dkey-103e21.5 | dre:562329/dre:566185/dre:394162/dre:561021 | 4 |
| dre01212 | Fatty acid metabolism | 5/511 | 91/8379 | 0.6589711 | 0.9630317 | 140615/393441/559403\_1/554145/393657 | fads2/hacd3/acaca/elovl8b/zgc:66024 | dre:140615/dre:393441/dre:559403/dre:554145/dre:393657 | 5 |
| dre04261 | Adrenergic signaling in cardiomyocytes | 17/511 | 301/8379 | 0.6658195 | 0.9630317 | 323509\_1/336121/562552/556410\_1/793666\_1/100535196/568726/555423\_1/677750/794522/559392\_1/562282/114457\_1/560807/492486/100538072/558525\_1 | gnaia/calm2a/zgc:171775/atf4b/popdc2/cacna2d3a/adcy7/scn1ba/myl2b/si:ch211-139a5.9/ppp2r2cb/ppp2r5b/atp1b2b/adcy6b/adra1bb/creb3l3b/atp2b3b | dre:323509/dre:192322/dre:562552/dre:556410/dre:793666/dre:100535196/dre:568726/dre:555423/dre:677750/dre:794522/dre:559392/dre:562282/dre:114457/dre:560807/dre:492486/dre:100538072/dre:558525 | 17 |
| dre04218 | Cellular senescence | 13/511 | 234/8379 | 0.6774775 | 0.9630317 | 333941\_1/336121/393186\_1/431763/562552/767751\_1/100000252\_3/393176/557551\_1/100330617\_1/565241/100000252/559723 | zfp36l2/calm2a/zgc:56235/gadd45ab/zgc:171775/ccnb3/pik3r3b/rad9a/hipk2/tsc1b/ppp3ccb/pik3r3b/tgfb2l | dre:333941/dre:192322/dre:393186/dre:431763/dre:562552/dre:767751/dre:100000252/dre:393176/dre:557551/dre:100330617/dre:565241/dre:100000252/dre:559723 | 13 |
| dre04114 | Oocyte meiosis | 10/511 | 182/8379 | 0.6796970 | 0.9630317 | 336121/562552/568726/562282/565241/560807/245702/100009657\_1/100005148\_1/280649\_1 | calm2a/zgc:171775/adcy7/ppp2r5b/ppp3ccb/adcy6b/igf1rb/rps6ka3b/ar/plk1 | dre:192322/dre:562552/dre:568726/dre:562282/dre:565241/dre:560807/dre:245702/dre:100009657/dre:100005148/dre:280649 | 10 |
| dre00534 | Glycosaminoglycan biosynthesis - heparan sulfate / heparin | 2/511 | 38/8379 | 0.6832609 | 0.9630317 | 792524/791107\_1 | hs6st3a/hs6st1b | dre:792524/dre:791107 | 2 |
| dre00250 | Alanine, aspartate and glutamate metabolism | 3/511 | 57/8379 | 0.6843252 | 0.9630317 | 334431\_1/447815/565235\_1 | adsl/rimkla/aldh5a1 | dre:334431/dre:447815/dre:565235 | 3 |
| dre04744 | Phototransduction | 3/511 | 57/8379 | 0.6843252 | 0.9630317 | 336121/792319/140425 | calm2a/saga/gc2 | dre:192322/dre:792319/dre:140425 | 3 |
| dre04622 | RIG-I-like receptor signaling pathway | 4/511 | 76/8379 | 0.6894278 | 0.9630317 | 562552/561021/393144\_2/100148871 | zgc:171775/si:dkey-103e21.5/trim25/dhx58 | dre:562552/dre:561021/dre:393144/dre:100148871 | 4 |
| dre03410 | Base excision repair | 3/511 | 58/8379 | 0.6957238 | 0.9630317 | 110438871/393774/556995\_1 | LOC110438871/pole3/lig1 | dre:110438871/dre:393774/dre:556995 | 3 |
| dre00604 | Glycosphingolipid biosynthesis - ganglio series | 1/511 | 19/8379 | 0.6978695 | 0.9630317 | 394230 | st3gal5 | dre:394230 | 1 |
| dre00410 | beta-Alanine metabolism | 2/511 | 41/8379 | 0.7231695 | 0.9630317 | 541429/368239 | aoc2/aldh2.2 | dre:541429/dre:368239 | 2 |
| dre04136 | Autophagy - other | 2/511 | 41/8379 | 0.7231695 | 0.9630317 | 326974\_1/406843\_1 | gabarapa/igbp1 | dre:326974/dre:406843 | 2 |
| dre04371 | Apelin signaling pathway | 12/511 | 226/8379 | 0.7323144 | 0.9630317 | 323509\_1/117508\_1/336121/30498\_1/117508/321449/100331581/568726/677750/560807/557486\_1/335655\_1 | gnaia/klf2a/calm2a/egr1/klf2a/ctgfa/si:dkey-44g17.6/adcy7/myl2b/adcy6b/hdac5/gngt2a | dre:323509/dre:117508/dre:192322/dre:30498/dre:117508/dre:321449/dre:100331581/dre:568726/dre:677750/dre:560807/dre:557486/dre:335655 | 12 |
| dre03020 | RNA polymerase | 2/511 | 42/8379 | 0.7354963 | 0.9630317 | 562329/394162\_1 | polr3b/polr3f | dre:562329/dre:394162 | 2 |
| dre04530 | Tight junction | 16/511 | 300/8379 | 0.7482572 | 0.9630317 | 57934\_1/337226\_1/286739/436718/798632/793158\_1/792828\_1/677750/556226\_1/793143/797651\_1/559392\_1/431777/793952\_1/100005566/101882911 | actb1/zgc:153867/msna/rhocb/myl6/cdc42l/pard6a/myl2b/cdc42l2/si:dkey-98f17.3/jam3a/ppp2r2cb/tuba7l/si:dkey-253i9.4/f11r.2/LOC101882911 | dre:57934/dre:337226/dre:286739/dre:436718/dre:798632/dre:793158/dre:792828/dre:677750/dre:556226/dre:793143/dre:797651/dre:559392/dre:431777/dre:793952/dre:100005566/dre:321070 | 16 |
| dre04110 | Cell cycle | 12/511 | 229/8379 | 0.7484883 | 0.9630317 | 560751\_1/431763/566841/327226/767751\_1/554509\_1/100141368\_1/794108\_1/562282/327060/280649\_1/559723 | stag2b/gadd45ab/crebbpa/casc5/ccnb3/orc1/waplb/nipblb/ppp2r5b/wee1/plk1/tgfb2l | dre:560751/dre:431763/dre:566841/dre:327226/dre:767751/dre:554509/dre:100141368/dre:794108/dre:562282/dre:327060/dre:280649/dre:559723 | 12 |
| dre04620 | Toll-like receptor signaling pathway | 6/511 | 121/8379 | 0.7555531 | 0.9630317 | 562552/100000252\_3/394198/570238/561021/100000252 | zgc:171775/pik3r3b/fosab/fosaa/si:dkey-103e21.5/pik3r3b | dre:562552/dre:100000252/dre:394198/dre:570238/dre:561021/dre:100000252 | 6 |
| dre00430 | Taurine and hypotaurine metabolism | 1/511 | 23/8379 | 0.7652495 | 0.9630317 | 794311 | fmo5 | dre:794311 | 1 |
| dre00071 | Fatty acid degradation | 3/511 | 65/8379 | 0.7668796 | 0.9630317 | 116517/368239/393860 | adh5/aldh2.2/gcdha | dre:116517/dre:368239/dre:393860 | 3 |
| dre04540 | Gap junction | 8/511 | 160/8379 | 0.7680901 | 0.9630317 | 323509\_1/100006675/568726/796490\_2/431777/560807/103909601/559806 | gnaia/prkcba/adcy7/pdgfbb/tuba7l/adcy6b/LOC103909601/htr2ab | dre:323509/dre:100006675/dre:568726/dre:796490/dre:431777/dre:560807/dre:103909601/dre:559806 | 8 |
| dre00830 | Retinol metabolism | 5/511 | 104/8379 | 0.7686189 | 0.9630317 | 116517/562840/553969/445083/393580 | adh5/rdh10a/cyp3a65/dhrs3a/bco1l | dre:116517/dre:562840/dre:553969/dre:445083/dre:393580 | 5 |
| dre03022 | Basal transcription factors | 2/511 | 45/8379 | 0.7697010 | 0.9630317 | 798997\_1/798997 | gtf2a2/gtf2a2 | dre:798997/dre:798997 | 2 |
| dre00982 | Drug metabolism - cytochrome P450 | 4/511 | 85/8379 | 0.7701702 | 0.9630317 | 116517/393493/431762\_1/794311 | adh5/gstt2/mgst1.2/fmo5 | dre:116517/dre:393493/dre:431762/dre:794311 | 4 |
| dre00053 | Ascorbate and aldarate metabolism | 3/511 | 66/8379 | 0.7758523 | 0.9630317 | 403070\_1/368239/445326\_1 | rgn/aldh2.2/akr1a1a | dre:403070/dre:368239/dre:445326 | 3 |
| dre03420 | Nucleotide excision repair | 3/511 | 68/8379 | 0.7929476 | 0.9630317 | 393774/556995\_1/100006257 | pole3/lig1/cetn2 | dre:393774/dre:556995/dre:100006257 | 3 |
| dre00563 | Glycosylphosphatidylinositol (GPI)-anchor biosynthesis | 1/511 | 25/8379 | 0.7930849 | 0.9630317 | 561471\_1 | pigl | dre:561471 | 1 |
| dre00670 | One carbon pool by folate | 1/511 | 25/8379 | 0.7930849 | 0.9630317 | 557552 | zgc:153031 | dre:557552 | 1 |
| dre04621 | NOD-like receptor signaling pathway | 10/511 | 205/8379 | 0.8104076 | 0.9631776 | 393186\_1/562552/436718/326974\_1/569298/566185/557239\_1/561021/110437957/110438502 | zgc:56235/zgc:171775/rhocb/gabarapa/ctsbb/caspbl/antxr2a/si:dkey-103e21.5/LOC110437957/LOC110438502 | dre:393186/dre:562552/dre:436718/dre:326974/dre:569298/dre:566185/dre:557239/dre:561021/dre:373874/dre:796505 | 10 |
| dre00531 | Glycosaminoglycan degradation | 1/511 | 27/8379 | 0.8176255 | 0.9631776 | 567720 | idua | dre:567720 | 1 |
| dre00970 | Aminoacyl-tRNA biosynthesis | 2/511 | 51/8379 | 0.8266973 | 0.9631776 | 101884223/792787 | LOC101884223/aars2 | dre:101884223/dre:792787 | 2 |
| dre00061 | Fatty acid biosynthesis | 1/511 | 28/8379 | 0.8287837 | 0.9631776 | 559403\_1 | acaca | dre:559403 | 1 |
| dre00511 | Other glycan degradation | 1/511 | 28/8379 | 0.8287837 | 0.9631776 | 566517 | aga | dre:566517 | 1 |
| dre04216 | Ferroptosis | 3/511 | 73/8379 | 0.8309767 | 0.9631776 | 796322/393186\_1/450084\_1 | slc3a2a/zgc:56235/gss | dre:796322/dre:393186/dre:450084 | 3 |
| dre00520 | Amino sugar and nucleotide sugar metabolism | 3/511 | 74/8379 | 0.8378208 | 0.9638088 | 100885772/321224/751668 | cmasb/hkdc1/gck | dre:100885772/dre:321224/dre:751668 | 3 |
| dre04217 | Necroptosis | 10/511 | 215/8379 | 0.8529883 | 0.9727778 | 110438871/393444/336426/393186\_1/337413\_1/566185/768248/561021/100149436/100148591 | LOC110438871/pygmb/chmp1b/zgc:56235/stat6/caspbl/fas/si:dkey-103e21.5/si:ch211-189a15.5/LOC100148591 | dre:110438871/dre:393444/dre:336426/dre:393186/dre:337413/dre:566185/dre:768248/dre:561021/dre:100149436/dre:561187 | 10 |
| dre00120 | Primary bile acid biosynthesis | 1/511 | 31/8379 | 0.8583333 | 0.9727778 | 641477 | zgc:123299 | dre:553543 | 1 |
| dre04141 | Protein processing in endoplasmic reticulum | 11/511 | 241/8379 | 0.8775049 | 0.9811648 | 386590\_1/327197\_1/266757/573376/337190/567029\_1/566876/556410\_1/567021/108181182/574002 | hsp90b1/vcp/tram1/hspa8/ssr3/rrbp1b/wfs1a/atf4b/ern2/LOC108181182/zgc:114174 | dre:386590/dre:327197/dre:266757/dre:573376/dre:337190/dre:567029/dre:566876/dre:556410/dre:567021/dre:567029/dre:574002 | 11 |
| dre04020 | Calcium signaling pathway | 22/511 | 450/8379 | 0.8883944 | 0.9811648 | 336121/393186\_1/368717/544664\_1/100006675/568726/796490\_2/494456/58147/101886891/565241/58133/492486/794365/100005590/103909601/445226/558525\_1/555941/559806/569706\_1/100137117\_1 | calm2a/zgc:56235/itpka/erbb3b/prkcba/adcy7/pdgfbb/fgf23/p2rx3a/LOC101886891/ppp3ccb/ngfb/adra1bb/si:dkey-163m14.7/hrh2b/LOC103909601/casq1a/atp2b3b/cacna1ba/htr2ab/phkb/si:dkey-1h24.2 | dre:192322/dre:393186/dre:368717/dre:100149258/dre:100006675/dre:568726/dre:796490/dre:494456/dre:58147/dre:101886891/dre:565241/dre:58133/dre:492486/dre:794365/dre:100005590/dre:103909601/dre:445226/dre:558525/dre:555941/dre:559806/dre:569706/dre:100137117 | 22 |
| dre04012 | ErbB signaling pathway | 5/511 | 127/8379 | 0.8941355 | 0.9811648 | 544664\_1/100000252\_3/100006675/556277/100000252 | erbb3b/pik3r3b/prkcba/cblb/pik3r3b | dre:100149258/dre:100000252/dre:100006675/dre:556277/dre:100000252 | 5 |
| dre00980 | Metabolism of xenobiotics by cytochrome P450 | 3/511 | 85/8379 | 0.8985776 | 0.9811648 | 116517/393493/431762\_1 | adh5/gstt2/mgst1.2 | dre:116517/dre:393493/dre:431762 | 3 |
| dre00040 | Pentose and glucuronate interconversions | 2/511 | 62/8379 | 0.8992447 | 0.9811648 | 445326\_1/393350 | akr1a1a/xylb | dre:445326/dre:393350 | 2 |
| dre00190 | Oxidative phosphorylation | 7/511 | 171/8379 | 0.9042107 | 0.9811648 | 447914\_1/573619/359840/767750\_2/447942/767750/359839 | cox17/ndufab1a/atp6v1b2/atp5ib/cox6a2/atp5ib/atp6v1ba | dre:447914/dre:573619/dre:359840/dre:767750/dre:447942/dre:767750/dre:359839 | 7 |
| dre03010 | Ribosome | 6/511 | 161/8379 | 0.9336941 | 0.9844026 | 336712\_2/415196\_1/327089/553284\_1/560828\_1/337007\_1 | rpl10/rpl5b/rpl12/mrpl1/rpl13a/rps12 | dre:336712/dre:415196/dre:327089/dre:553284/dre:560828/dre:337007 | 6 |
| dre04814 | Motor proteins | 13/511 | 305/8379 | 0.9378301 | 0.9844026 | 57934\_1/337226\_1/798632/101882034/474327/677750/556023\_1/799461\_2/431777/30429/571487/562052\_1/100334351 | actb1/zgc:153867/myl6/LOC101882034/capza1b/myl2b/dnah2/dnah12/tuba7l/mylpfa/si:dkeyp-92c9.3/kif14/dnah3 | dre:57934/dre:337226/dre:798632/dre:406297/dre:474327/dre:677750/dre:556023/dre:799461/dre:431777/dre:30429/dre:571487/dre:562052/dre:100334351 | 13 |
| dre03040 | Spliceosome | 6/511 | 164/8379 | 0.9405332 | 0.9844026 | 100004402\_1/100037380\_3/573376/100005893/566017\_1/334283\_2 | plrg1/zgc:163098/hspa8/magoh/lsm8/ddx23 | dre:100004402/dre:100037380/dre:573376/dre:100005893/dre:566017/dre:334283 | 6 |
| dre00860 | Porphyrin metabolism | 2/511 | 74/8379 | 0.9455714 | 0.9844026 | 100002875/101886332 | hmox2a/hephl1b | dre:100002875/dre:101886332 | 2 |
| dre04310 | Wnt signaling pathway | 11/511 | 278/8379 | 0.9573244 | 0.9844026 | 678649\_1/566841/436718/100000500\_1/100006675/564241/103909653/565241/556087/58072\_1/101882911 | zgc:136929/crebbpa/rhocb/sost/prkcba/fosl1a/LOC103909653/ppp3ccb/wnt2bb/fzd8b/LOC101882911 | dre:678649/dre:566841/dre:436718/dre:100000500/dre:100006675/dre:564241/dre:103909653/dre:565241/dre:556087/dre:58072/dre:321070 | 11 |
| dre04260 | Cardiac muscle contraction | 6/511 | 173/8379 | 0.9574350 | 0.9844026 | 100535196/447942/677750/556638\_2/794522/114457\_1 | cacna2d3a/cox6a2/myl2b/slc9a6b/si:ch211-139a5.9/atp1b2b | dre:100535196/dre:447942/dre:677750/dre:556638/dre:794522/dre:114457 | 6 |
| dre03250 | Viral life cycle - HIV-1 | 2/511 | 83/8379 | 0.9661439 | 0.9844026 | 566841/64275\_1 | crebbpa/supt5h | dre:566841/dre:64275 | 2 |
| dre04350 | TGF-beta signaling pathway | 5/511 | 161/8379 | 0.9719155 | 0.9844026 | 566841/436718/100004116\_1/405778/559723 | crebbpa/rhocb/fsta/grem1a/tgfb2l | dre:566841/dre:436718/dre:100004116/dre:100332300/dre:559723 | 5 |
| dre04144 | Endocytosis | 17/511 | 417/8379 | 0.9750131 | 0.9844026 | 792229\_1/573376/336426/406837\_1/436718/793158\_1/336368/406684\_1/792828\_1/393881/474327/556226\_1/556277/565743\_1/568170\_1/561771/245702 | sh3glb1a/hspa8/chmp1b/rnf41/rhocb/cdc42l/vps37a/vps26b/pard6a/vps26a/capza1b/cdc42l2/cblb/pld2/eps15l1a/acap1/igf1rb | dre:792229/dre:573376/dre:336426/dre:406837/dre:436718/dre:793158/dre:336368/dre:406684/dre:792828/dre:393881/dre:474327/dre:556226/dre:556277/dre:565743/dre:568170/dre:561771/dre:245702 | 17 |
| dre00600 | Sphingolipid metabolism | 2/511 | 89/8379 | 0.9754554 | 0.9844026 | 450083/493602 | gla/asah2 | dre:450083/dre:493602 | 2 |
| dre00140 | Steroid hormone biosynthesis | 2/511 | 91/8379 | 0.9779686 | 0.9844026 | 553969/791578\_1 | cyp3a65/dhrs11a | dre:553969/dre:791578 | 2 |
| dre04920 | Adipocytokine signaling pathway | 2/511 | 108/8379 | 0.9913296 | 0.9913296 | 406473/100149042 | pck2/agrp | dre:406473/dre:100149042 | 2 |
